# On geometry parameterization for simulation-driven design closure of antenna structures

**DOI:** 10.1038/s41598-021-03728-4

**Published:** 2021-12-21

**Authors:** Slawomir Koziel, Anna Pietrenko-Dabrowska

**Affiliations:** 1 Engineering Optimization and Modeling Center, Reykjavik University, 102 Reykjavík, Iceland; 2 Faculty of Electronics, Telecommunications and Informatics, Gdansk University of Technology, 80-233 Gdańsk, Poland

**Keywords:** Electrical and electronic engineering, Computational science

## Abstract

Full-wave electromagnetic (EM) simulation tools have become ubiquitous in antenna design, especially final tuning of geometry parameters. From the reliability standpoint, the recommended realization of EM-driven design is through rigorous numerical optimization. It is a challenging endeavor with the major issues related to the high computational cost of the process, but also the necessity of handling several objectives and constraints over often highly-dimensional parameter spaces. From the numerical perspective, making decisions about the formulation of the optimization problem, the approach to handling the design constraints, but also parameterization of the antenna geometry, are all non-trivial. At the same time, these issues are interleaved, and may play an important role in the performance and reliability of the simulation-based design closure process. This paper demonstrates that the approach to arranging the structure parameterization (e.g., the use of absolute or relative parameters) may have a major effect of the optimization outcome. Our investigations are carried out using three broadband monopole antennas optimized under different scenarios and using different parameterizations. In particular, the results indicate that relative parameterization is preferred for optimization of input characteristics, whereas absolute parameterization is more suitable for size reduction.

## Introduction

The last decade or so has witnessed a significant increase in the complexity of antenna geometries^1–5^. This can be mainly attributed to the emergence of new technologies (4G and 5G wireless communications^6^, medical imaging^7^, wireless sensing^8^, implantable devices^9^), but also trends towards miniaturization and portability (mobile communications^10^, internet of things, IoT^11^, wearable devices^12^). On the one hand, to fulfil the performance requirements imposed by the needs pertinent to specific application areas, antenna structures have to implement additional functionalities (multi-band operation^13^, tunability^14^, circular polarization^15^), but also offer improved characteristics (high gain^16^, broadband operation^17^, high element isolation in MIMO systems^18^, etc.). This requires incorporation of a variety of geometrical alterations (stubs^19^, slots^20^, defected ground structures^21^, metamaterial-based components^22^), which makes the designs increasingly sophisticated and described by large numbers of parameters. Also, the use of full-wave electromagnetic (EM) simulation tools is instrumental in ensuring evaluation reliability. On the other hand, miniaturization trends lead to additional challenges as size reduction is detrimental to antenna electrical properties^23,24^. In any case, EM-driven design becomes a matter of practical necessity in general, whereas simulation-based parameter tuning belongs to the most important stages of the antenna design cycle.

EM-simulation-based parameter tuning of antenna systems is imperative, yet it is a daunting task. Its fundamental bottlenecks include high computational cost, a typically large number of parameters, but also the necessity of handling several objectives and constraints. Over the years, the literature offered a variety of algorithmic solutions that aim at alleviating these difficulties. Some of available options include methods for expediting the EM-driven optimization procedures, e.g., adjoint sensitivities^25^, sparse Jacobian updates^26^, surrogate-assisted methods, involving both data-driven (kriging^27^, radial-basis functions^28^, neural networks^29^, Gaussian process regression^30^, ensemble learning methods^31^), and physics-based models (space mapping^32^, manifold mapping^33^, response correction methods^34,35^, cognition-driven design^36^), or machine learning techniques^37,38^. Handling of multiple objectives is often realized using surrogate-enhanced population-based methods^39–41^ or penalty function approaches (e.g., for size reduction under multiple constraints imposed on antenna electrical performance^42^), whereas dimensionality issues are often addressed using high-dimensional model representation (HDMR)^43^, principal component analysis (PCA)^44^, model order reduction methods^45^, or—in the context of response surface approximation—techniques such as orthogonal matching pursuit (OMP)^46^, or least angle regression (LAR)^47^. Surrogate-based methods are also popular for aiding global optimization^48,49^. The improvement of local search reliability (e.g., under the lack of quality initial designs) can be achieved using feature-based optimization (FBO)-type of approaches^50,51^. Accelerating specific simulation-driven design tasks can be realized using particular classes of replacement models (e.g., polynomial chaos expansion, PCE, for uncertainty quantification^52,53^).

One of the important aspects of antenna optimization in general, and EM-driven design in particular, is appropriate geometry parameterization of the structure under design. As a matter of fact, this aspect is almost never explicitly discussed in the literature, although parameterization may have some serious implications for the design process reliability. In the vast majority of cases, a natural (or absolute) parameterization is employed, where specific dimensions of the antenna components (radiator width and length, stub lengths, slot distance, ground plane length, etc.) are described using respective variables, typically sized in millimeters. From the perspective of numerical optimization, the fundamental problem incurred by such an approach are design constraints that have to be imposed in order to ensure geometrical consistency of the structure, e.g., to have the radiator allocated within the dielectric substrate outline, the ground-plane slots contained within the ground plane rather than cutting it into disjoint patches, etc. Most of these constraints are linear, although nonlinear ones may also be necessary, e.g., to ensure that certain components are allocated in the interior of circular-shaped slots, etc. The presence of constraints makes the optimization problem more challenging, which is of particular importance when handling EM-simulated antenna responses. The latter is due to the numerical noise inherent to simulated characteristics of the device. A less popular alternative is relative parameterization, where the antenna components are sized in relation to the dimensions of the components encapsulating them (e.g., a radiator slot length being a fraction the radiator size, or substrate size being a sum of encapsulated components such as the feed line length, radiator length, etc.). A clear advantage is that the number of geometrical constraints can be reduced to a minimum (often to zero), which leads to an interval-based parameter space, significantly easier to handle.

This paper investigates the importance of geometry parameterization from the perspective of EM-driven tuning of antenna structures. Two types of parameterization are juxtaposed, referred to the absolute and the relative ones (as outlined in the previous paragraph), along with their potential advantages and drawbacks from the point of view of solving antenna optimization tasks. Using several exemplary structures of broadband antennas, it is demonstrated that relative parameterization is more suitable for improving electrical properties (primarily, the input characteristics). On the other hand, the absolute parameterization is more beneficial for explicit size reduction, which is a constrained task by itself due to the necessity of maintaining specific acceptance levels for input matching, and, perhaps, other antenna responses. Consistency of the results throughout the benchmark set as well as considerable differences in the optimization process performance, provide conclusive evidence about the importance of the appropriate choice of antenna parameterization within a given EM-based design context.

The originality and the technical contributions of this work can be summarized as follows: (1) formal introduction of absolute and relative antenna parameterizations, (2) qualitative comparison of the benefits and limitations of the absolute and relative parameterizations in the context of different EM-driven design scenarios, (3) comprehensive (based on three antenna structures and multiple optimization runs) and conclusive assessment of the advantages of relative parameterization for matching improvement tasks, and absolute one for size reduction purposes. According to the authors’ knowledge, this is the first treatment of this subject (both qualitative and quantitative) in the antenna design optimization literature.

### Simulation-driven design of antenna structures: geometry parameterization

This section formally introduces the two types of antenna geometry parameterizations considered in this paper, and discusses their qualitative advantages and disadvantages from the point of view of solving EM-driven design tasks. Quantitative evaluation, based on three antenna structures and two specific design scenarios (size reduction and matching improvement), will be provided in “Results”.

### Antenna parameter tuning: problem formulation

The computational model of the antenna structure under design will be denoted as ***R***, where ***x*** = [*x*_1_ … *x*_*n*_]^*T*^ is the vector of (independent) adjustable parameters. ***R*** is assumed to be evaluated using full-wave EM analysis. ***R***(***x***) will represent all relevant antenna characteristics at the design ***x***, in particular, the reflection response *S*_11_(***x***,*f*), gain *G*(***x***,*f*), axial ratio *AR*(***x***,*f*), etc., for frequencies *f* within the simulation range of interest. Furthermore, we will denote the antenna size as *A*(***x***) (e.g., the footprint area in the case of planar antennas).

The parameter tuning task is formulated as a minimization problem

1 $${\mathbf{x}}^{*} = \arg \mathop {\min }\limits_{{{\mathbf{x}} \in X}} U({\mathbf{x}})$$

where *X* is the problem domain, discussed at length in “Geometry parameterization: Absolute vs. relative”. In some cases, additional constraints are imposed, which are typically of an inequality type, i.e., *g*_*k*_(***x***) ≤ 0, *k* = 1, …, *n*_*g*_.

The objective function is a metric of the design quality, and, therefore, it is problem dependent. Consider the following examples:

- Matching improvement in a specified frequency range *F*: *U*(***x***) = max{*f* ∈ *F* : |*S*_11_(***x***,*f*)|};
- Axial ratio improvement in a specified frequency range *F*: *U*(***x***) = max{*f* ∈ *F* : *AR*(***x***,*f*)};
- Size reduction of a planar antenna: *U*(***x***) = *A*(***x***)

Design constraints are often related to antenna geometry itself (cf. “Geometry parameterization: Absolute vs. relative”) but also its electrical and field performance figures. Some common examples include:

- Ensuring that antenna reflection does not exceed –10 dB within the frequency range of interest *F*: *S*(***x***) ≤ –10 dB, where *S*(***x***) = max{*f* ∈ *F* : |*S*_11_(***x***,*f*)|};
- Ensuring that axial ratio does not exceed 3 dB within the frequency range of interest *F*: *A*_*R*_(***x***) ≤ 3 dB, where *A*_*R*_(***x***) = max{*f* ∈ *F* : *AR*(***x***,*f*)}.

As the constraints are often expensive to evaluate (i.e., require EM simulation of the antenna structure), their handling is more convenient when using a penalty function approach^54^. This leads to a problem reformulation, so that we have

2 $${\mathbf{x}}^{*} = \arg \mathop {\min }\limits_{{\mathbf{x}}} U_{P} ({\mathbf{x}})$$

where the objective function *U*_*P*_ is defined as

3 $$U_{P} ({\mathbf{x}}) = U({\mathbf{x}}) + \sum\nolimits_{k = 1}^{{n_{g} }} {\beta_{k} c_{k} ({\mathbf{x}})}$$

The second term in (3) is a linear combination of the penalty functions *c*_*k*_(***x***) quantifying violations of the respective constraints; *β*_*k*_ are the proportionality (penalty) coefficients. In order to make the objective function setup less dependent on a constraint type and typical tolerance levels, penalty functions may be defined based on relative violations, e.g., for the constraint *S*(***x***) ≤ –10 dB, one may define *c*(***x***) = [(*S*(***x***) + 10)/10]^2^.

### Geometry parameterization: absolute versus relative

The problem of antenna parameterization is rarely elaborated on in the literature. As a matter of fact, it is one of those aspects of the design process that are usually considered unimportant or straightforward to handle. In practice, it is normally reduced to selecting the crucial dimensions (usually, by means of initial parametric studies), and labelling them accordingly for further processing. The purpose of this work is to indicate the relevance of appropriate handling of antenna parameterization as well as far reaching consequences of inappropriate parameter space definition.

We start by describing the two types of antenna parameterization, referred to as absolute and relative. The absolute parameterization corresponds to what is normally considered in practical antenna design, and, therefore, can also be named a natural one. According to this method, all relevant antenna parameters are simply assigned the labels, and processed (e.g., by the optimization procedures) using absolute dimensions expressed in appropriate units (e.g., millimetres).

**Figure 1:**
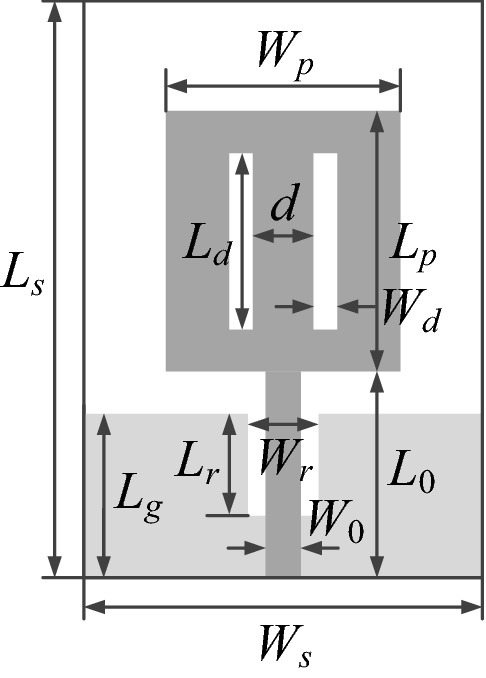
Absolute parameterization for an exemplary monopole antenna^55^ (ground plane marked using the light-shade grey). The geometry is described by the variable vector ***x*** = [*L*_*s*_* L*_*0*_* L*_*p*_* L*_*d*_* L*_*r*_* L*_*g*_* W*_*s*_* W*_*r*_* W*_*p*_* W*_*d*_* d*]^*T*^; variable *W*_0_ is normally fixed and adjusted for a given substrate to ensure 50-Ω line impedance. (Microsoft Visio 2016, https://www.microsoft.com/pl-pl/microsoft-365/visio/).

Figure 1 shows an example of a monopole antenna with radiator slots and a ground plane slot with all its dimensions labelled accordingly. In this case, the absolute parameterization is in one-to-one correspondence with these labels. Typically, all parameters have assigned their lower and upper bounds, which define the parameter space *X*. However, in order to maintain the geometrical consistency of the structure one has to impose additional constraints. For example, we have to introduce *L*_*d*_ < *L*_*p*_ (i.e., the radiator slots are contained within the radiator), *L*_*r*_ < *L*_*g*_ (the ground plane slot does not split the ground place into disjoint parts), or *L*_0_ + *L*_*p*_ ≤ *L*_*s*_ (the radiator is contained within the substrate). The full list of the necessary constraints can be found in Table 1. For the antenna of Fig. 1, we have six linear constraints in total, which makes the optimization task more challenging when using this parameterization. These have to be considered in addition to the constraints imposed on antenna characteristics (cf. “Antenna parameter tuning. Problem formulation”).

**Table 1:** Absolute versus relative parameterization for the monopole antenna of Fig. 1.

Characteristic	Parameterization	
	Absolute	Relative
Independent parameters	***x*** = [*L*_*s*_* L*_0_ *L*_*p*_* L*_*d*_* L*_*r*_* L*_*g*_* W*_*s*_* W*_*r*_* W*_*p*_* W*_*d*_* d*]^*T*^	***x*** = [*L*_*s*_* L*_0*r*_* L*_*pr*_* L*_*dr*_* L*_*rr*_* L*_*gr*_* W*_*s*_* W*_*rr*_* W*_*pr*_* W*_*d*_* d*_*r*_]^*T*^
Box constraints	*L*_*s.*min_ ≤ *L*_*s*_ ≤ *L*_*s.*max_; *L*_0*.*min_ ≤ *L*_0_ ≤ *L*_0*.*max_; *L*_*p.*min_ ≤ *L*_*p*_ ≤ *L*_*p.*max_; … *W*_*d.*min_ ≤ *W*_*d*_ ≤ *W*_*d.*max_; *d*_min_ ≤ *d* ≤ *d*_max_	*L*_*s.*min_ ≤ *L*_*s*_ ≤ *L*_*s.*max_; *W*_*s.*min_ ≤ *W*_*s*_ ≤ *W*_*s.*max_; 0 ≤ *L*_0*r*_, *L*_*pr*_, *L*_*dr*_, *L*_*rr*_, *L*_*gr*_ ≤ 1; 0 ≤ *W*_*rr*_, *W*_*pr*_, *d*_*r*_ ≤ 1; *W*_*d.*min_ ≤ *W*_*d*_ ≤ *W*_*d.*max_
Parameter space *X*	[*L*_*s.*min_ *L*_*s.*max_] × [*L*_0*.*min_ *L*_0*.*max_] × … … × [*W*_*d.*min_ *W*_*d.*max_] × [*d*_min_ *d*_max_]	[*L*_*s.*min_ *L*_*s.*max_] × [0 1] × … × [0 1] × [*W*_*s.*min_ *W*_*s.*max_] × [0 1] × [0 1] × [0 1] × [*W*_*d*.min_ *W*_*d*.max_] × [0 1]
Additional constraints	*L*_*d*_ ≤ *L*_*p*_; *d* + 2*W*_*d*_ ≤ *W*_*p*_; *L*_0_ + *L*_*p*_ ≤ *L*_*s*_; *L*_*g*_ ≤ *L*_*s*_; *W*_*r*_ ≤ *W*_*s*_; *W*_*p*_ ≤ *W*_*s*_	None
Additional relationships	None	*L*_0_ = *L*_*s*_*L*_0*r*_; *L*_*p*_ = (*L*_*s*_ − *L*_0_)*L*_*pr*_; *L*_*d*_ = *L*_*p*_*L*_*dr*_; *L*_*r*_ = *L*_*g*_*L*_*rr*_; *L*_*g*_ = *L*_*s*_*L*_*gr*_; *W*_*r*_ = *W*_*s*_*W*_*rr*_; *W*_*p*_ = *W*_*s*_*W*_*pr*_;*d* = (*W*_*p*_ − 2*W*_*d*_)*d*_*r*_

Relative parameterization can be introduced to reduce the number of additional geometry constraints. According to it, most of the parameters are used to dimension particular antenna components in relation to the containing elements (e.g., the radiator slot length with respect to the radiator length, etc.). Only the outer dimensions of the antenna as well as parameters that are inherently small (e.g., slot widths) are treated as absolute. Table 1 contains a full description of this parameterization. It can be noted that a certain number of additional relationships is required (to evaluate the absolute antenna dimensions), but no additional constraints are necessary.

In other words, the parameter space is an interval, which is much easier to handle by local search procedures, let alone nature-inspired algorithms (where constrained optimization is a non-trivial task^56–58^).

It should be noticed that relative parameterization can be also arranged by introducing alternative absolute parameters such as *d*_*L*_ or *d*_*W*_ so that we have *L*_*s*_ = *L*_0_ + *L*_*p*_ + *d*_*L*_ and *W*_*s*_ = *W*_*p*_ + 2*d*_*W*_, in which case the parameters *L*_0_, *L*_*p*_, and *W*_*p*_ can be kept absolute, while retaining the overall advantages of the relative parameterization, i.e., the lack of additional constraints, and the parameter space being the interval.

### Qualitative comparison of antenna parameterizations

This section discusses some of the basic properties of the two types of antenna parameterization considered in “Geometry parameterization: Absolute vs. relative”, along with potential implication for solving antenna optimization tasks. These are gathered in Table 2. Perhaps the biggest advantage of relative parameterization is the simplicity of the parameter space, which is an interval. Normally, no additional geometry constraints are necessary, which simplifies handling of the parameters, and increases the range of optimization algorithms that can be employed. On the other hand, absolute parameterization is simpler in terms of providing a direct account for antenna dimensions (one-to-one correspondence between design variables and antenna dimensions). It seems that this might be beneficial for solving tasks such as explicit miniaturization, where antenna dimensions directly contribute to the definition of the objective function. At the same time, this might cause problems when handling objectives related to electrical characteristics, e.g., matching improvement, bandwidth enhancement, gain maximization, axial ratio minimization, etc. It should also be emphasized that the aforementioned potential problems are mainly a result of handling EM-simulated antenna responses, which contain a certain level of numerical noise^59^. The differences between the two parameterizations would most likely not be noticeable if inherently smooth objective functions and constraints were to be processed.

**Table 2:** Absolute versus relative parameterization: Basic properties.

Feature	Parameterization	
	Absolute	Relative
Parameter space and geometry constraints	Box constraints and (usually) linear constraints on antenna dimensions	Only lower and upper parameter bounds (box constraints)
Handling antenna dimensions	Direct: one-to-one correspondence between parameters and antenna dimensions	Indirect: some dimensions calculated using relative and absolute parameters
**Solving optimization tasks**		
Pros	Direct account for antenna dimensions (suitable for size reduction tasks)	Reduced number of geometry constraints
Cons	Potentially complex geometry of a feasible region (may lead to additional challenges when handling electrical and field characteristics)	Indirect relationship between design variables and antenna geometry (may not be suitable for size reduction tasks)

### Absolute versus relative parameterization: comparative study

The purpose of this section is a comparative study concerning utilization of the two types of geometry parameterization, absolute and relative, for solving antenna parameter tuning tasks. We consider two qualitatively different problems, optimization for minimum size, and optimization for best in-band matching. As mentioned before, these problems have entirely different characteristics, e.g., size reduction features smooth primary objective and expensive constraints, whereas reflection improvement task uses expensive (EM-evaluated) objective and geometry-only constraints. As demonstrated below, antenna parameterization is of paramount importance from the point of view of the optimization process performance.

### Benchmark antenna structures and parameterizations

**Figure 2:**
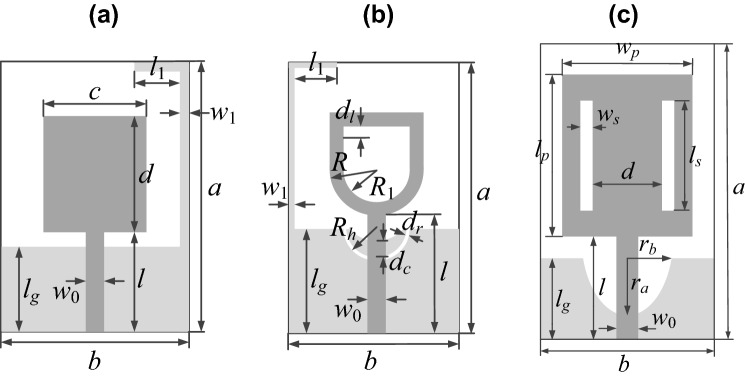
Benchmark antenna structures: **(a)** Antenna I^60^, **(b)** Antenna II^61^, **(c)** Antenna III^55^. Ground planes marked using light gray shade. (Microsoft Visio 2016, https://www.microsoft.com/pl-pl/microsoft-365/visio/).

The numerical studies are carried out using three broadband monopole antennas shown in Fig. 2. All structures are to operate within UWB band of 3.1 GHz to 10.6 GHz. Antennas I^60^ and II^61^ are implemented on RF-35 substrate (*ε*_*r*_ = 3.5, *h* = 0.762 mm), whereas Antenna III^55^ is implemented on FR4 substrate (*ε*_*r*_ = 4.3, *h* = 1.55 mm). The feed lines widths (parameter *w*_0_) are dimensioned to ensure 50-Ω input impedance: we have *w*_0_ = 1.7 mm for Antenna I and II, and *w*_0_ = 3.0 mm for Antenna III. The EM models are evaluated using time-domain solver of CST Microwave Studio. The models include the SMA connectors. In all cases, metallization if represented as perfect electrical conductor (PEC), whereas dielectric losses are taken into account.

For each antenna, we consider two parameterizations, the absolute and the relative one (cf. “Geometry parameterization: Absolute vs. relative”). The details concerning both parameterizations for Antenna I, II, and III are provided in Table 3, which also contains information about additional geometry constraints as well as the relationships between the antenna dimensions and design variables.

**Table 3:** Parameterization of Antenna I, II, and III (Fig. 2).

Antenna	Parameterization	
	Absolute	Relative
**I**		
Independent parameters	***x*** = [*a b l c d l*_*g*_* l*_1_ *w*_1_]^*T*^	***x*** = [*l c d d*_*l*_* d*_*w*_* l*_*gr*_* l*_1*r*_* w*_1_]^*T*^
Additional constraints	*l* + *d* ≤ *a*; *d* ≤ *b*; *l*_*g*_ ≤ *a* – *w*_1_; *l*_1_ + *w*_1_ ≤ *b*	None
Additional relationships	None	*a* = *l* + *d* + *d*_*l*_; *b* = 2*d*_*w*_ + *c*; *l*_*g*_ = *l*_*gr*_(*a* – *w*_1_); *l*_1_ = *l*_1*r*_(*b* – *w*_1_)
**II**		
Independent parameters	***x*** = [*a b l d*_*l*_* R R*_1_ *l*_*g*_* l*_1_ *R*_*h*_* d*_*r*_* d*_*c*_* w*_1_]^*T*^	***x*** = [*l d*_*l*_* R R*_1*r*_* d*_*a*_* d*_*b*_* l*_*gr*_* l*_1*r*_* R*_*hr*_* d*_*rr*_* d*_*cr*_* w*_1_]^*T*^
Additional constraints	*l* + 3*R* – *R*_1_ + *d*_*l*_ ≤ *a*; 2*R* ≤ *b*; *R*_1_ ≤ *R*; *d*_*r*_ ≤ *R*_*h*_; *w*_1_ + *l*_1_ ≤ *b*; *l*_*g*_ ≤ *a* – *w*_1_; *d*_*c*_ ≤ *R*_*h*_ – *d*_*r*_	None
Additional relationships	None	*R*_1_ = *R*_1*r*_*R*; *a* = *l* + 3*R* – *R*_1_ + *d*_*l*_ + *d*_*a*_; *l*_*g*_ = *l*_*gr*_(*a* – *w*_1_); *l*_1_ = *l*_1*r*_(*b* – *w*_1_); *R*_*h*_ = *R*_*hr*_*b*/2; *d*_*r*_ = *d*_*rr*_*R*_*h*_; *d*_*c*_ = *d*_*cr*_(*R*_*h*_ – *d*_*r*_) + *d*_*r*_
**III**		
Independent parameters	***x*** = [*a b l l*_*p*_* w*_*p*_* l*_*s*_* w*_*s*_* d l*_*g*_* r*_*a*_* r*_*b*_]^*T*^	***x*** = [*l*_*gr*_* l l*_*s*_* w*_*s*_* d d*_*a*_* d*_*ls*_* d*_*ws*_* d*_*w*_* r*_*ar*_* r*_*br*_]^*T*^
Additional constraints	*w*_*p*_ ≤ *b*; 2*w*_*s*_ + *d* ≤ *w*_*p*_; *l* + *l*_*p*_ ≤ *a*; *l*_*s*_ ≤ *l*_*p*_; 2*r*_*b*_ ≤ *b*; *r*_*a*_ ≤ *l*_*g*_	None
Additional relationships	None	*a* = *l* + *l*_*p*_ + *d*_*a*_; *b* = *d* + 2*w*_*s*_ + 2*d*_*ws*_ + 2*d*_*b*_; *l*_*p*_ = *l*_*s*_ + 2*d*_*ls*_; *w*_*p*_ = *d* + 2*w*_*s*_ + 2*d*_*ws*_; *l*_*g*_ = *l*_*gr*_*a*; *r*_*a*_ = *r*_*ar*_*l*_*g*_; *r*_*b*_ = *r*_*br*_*b*/2

The work focuses on establishing antenna parameterization for the purpose of design optimization. Thus, experimental validation of the designs of the considered antenna structures has not been provided as it is immaterial to the topic of the paper. Moreover, all of the considered structures have been already validated in the respective source papers^55,60,61^, but also in our previous works where their numerical optimization has been performed^49,62,63^.

### Numerical experiments: setup

In order to evaluate the merits of the considered antenna parameterizations, we carry out optimization of the structures of Fig. 2 under the following two scenarios:

- Optimization for matching improvement in the UWB frequency range, in which case, the objective function is of the form *U*(***x***) = max{3.1 GHz ≤ *f* ≤ 10.6 GHz : |*S*_11_(***x***,*f*)|};
- Optimization for size reduction; the objective function is of the form *U*(***x***) = *A*(***x***). Furthermore, the condition *S*(***x***) ≤ –10 dB is imposed on the reflection response, where *S*(***x***) = max{3.1 GHz ≤ *f* ≤ 10.6 GHz : |*S*_11_(***x***,*f*)|}. The constraint is handled implicitly as in (3) with the penalty function of the form *c*(***x***) = [(*S*(***x***) + 10)/10]^2^. For all considered structures, the size is understood as the area of the entire substrate containing the antenna.

The optimization algorithm of choice is trust-region gradient search with numerical derivatives^62^, which produces a series of approximations to the optimal design x^*^ using an auxiliary linear expansion model of antenna responses established at the iteration point. More details about the procedure can be found in the literature^63,64^.

For each design scenario, the optimization process is executed ten times from random initial designs (the same starting points are used in both scenarios). This is to account for the fact that the optimization problem might be multimodal due to a relatively large number of parameters as well as the presence of numerical noise. For both reasons, the final design generally depends on the starting point; consequently, evaluating performance of the algorithm based on a single run is not representative.

The following metrics are used:

- Optimization for matching improvement: maximum in-band reflection, the value averaged over ten algorithm runs as well as the standard deviation;
- Optimization for size reduction: obtained footprint area (average and standard deviation), as well as violation of the reflection constraint *S*(***x***) ≤ –10 dB (average value and the standard deviation);

Comparison of the aforementioned factors for both antenna parameterizations will provide meaningful assessment of the optimization process performance and reliability.

### Numerical experiments: results and discussion

**Figure 3:**
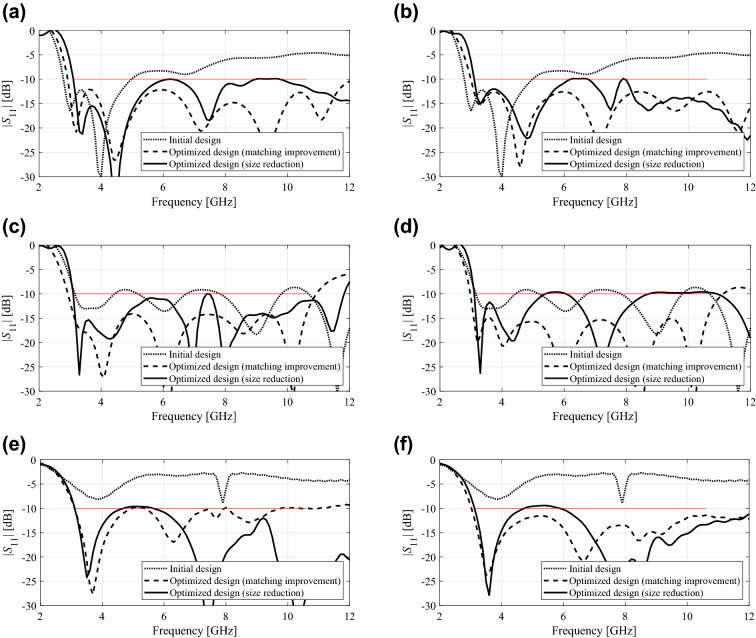
Selected optimization runs for Antennas I through III (see also Table 5): initial design (⋅⋅⋅⋅), design x^*1^ optimized for best matching, (- - -), design x^*2^ optimized for minimum size (—); Antenna I: **(a)** absolute parameterization (*S*(x^*1^) = − 12.4 dB, *A*(x^*2^) = 278 mm^2^), **(b)** relative parameterization (*S*(x^*1^) = − 12.2 dB, *A*(x^*2^) = 300 mm^2^); Antenna II: **(c)** absolute parameterization (*S*(x^*1^) = − 14.0 dB, *A*(x^*2^) = 225 mm^2^), **(d)** relative parameterization (*S*(x^*1^) = − 14.9 dB, *A*(x^*2^) = 293 mm^2^); Antenna III: **(e)** absolute parameterization (*S*(x^*1^) = − 10.0 dB, *A*(x^*2^) = 201 mm^2^), **(f)** relative parameterization (*S*(x^*1^) = − 11.4 dB, *A*(x^*2^) = 229 mm^2^). Design specifications for antenna reflection marked using a horizontal line. (Matlab R2016a https://www.mathworks.com).

**Figure 4:**
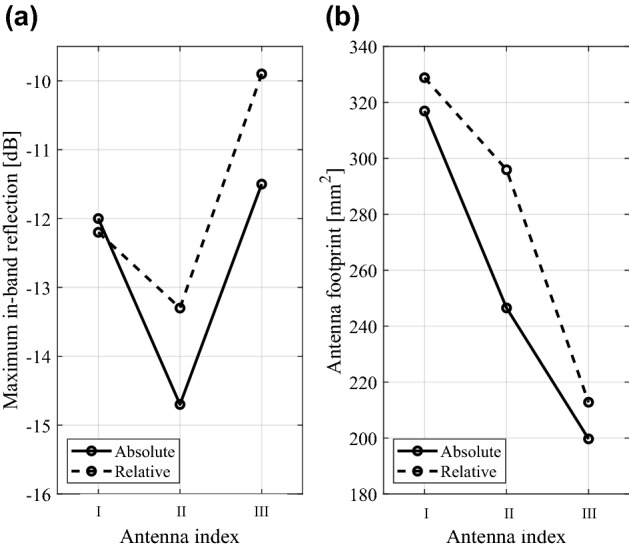
Comparison of the optimization process performance between the absolute (—) and relative (- - -) antenna parameterization. Shown are: **(a)** the maximum in-band reflection of the antenna, averaged over the ten independent optimization runs (optimization for matching improvement), and **(b)** antenna footprint area averaged over the ten independent optimization runs (optimization for size reduction). Consistent differences between the two parameterization can be observed, in favor of relative parameterization when optimizing for matching improvement, and absolute parameterization when optimizing for size reduction. (Matlab R2016a https://www.mathworks.com).

Table 4 provides the numerical results for Antennas I, II, and III, respectively. Furthermore, Fig. 3 illustrates the initial and optimized designs for the selected algorithm runs, whereas Table 5 gathers the respective geometry parameter vectors. Figure 4 shows the comparison of the average in-band reflection level and the footprint area for the considered antennas optimized using the absolute and relative parameterizations.

**Table 4:** Optimization results of Antennas I through III.

Antenna		I		II		III	
Optimization scenario	Performance metric	Parameterization					
		Absolute	Relative	Absolute	Relative	Absolute	Relative
Matching improvement	*U*(x*)^1^ (dB)	− 12.2	− 12.0	− 13.3	− 14.7	− 9.9	− 11.5
	Std[*U*(x*)] (dB)	0.8	0.6	0.6	0.5	1.8	0.5
	Cost^4^	88.5	89.4	126.1	126.3	139.9	146.3
Size reduction	Footprint area *A*(x*)^2^ (mm^2^)	316.9	328.8	246.5	295.9	199.7	212.8
	Std[*A*(x*)]	36.5	30.4	19.4	37.1	16.1	14.2
	Constraint violation *D*^3^ (dB)	0.9	0.9	0.4	1.0	0.9	0.9
	Std[D] (dB)	0.9	0.7	0.3	0.4	0.3	0.3
	Cost^4^	112.2	103.5	191.3	176.8	163.6	164.9

^1^Optimized maximum in-band reflection, averaged over ten algorithm runs.

^2^Optimized antenna footprint averaged over ten algorithm runs.

^3^Constraint violation, defined as *D* = {3.1 GHz ≤ *f* ≤ 10.6 GHz : max|*S*_11_(*f*)|} + 10, averaged over ten algorithm runs.

^4^Number of EM simulations averaged over ten algorithm runs.

**Table 5:** Optimized geometry parameter vectors for the designs of Fig. 3.

Antenna	Param-etrization	Design scenario	Geometry parameter values (mm)											
I	Absolute		*a*	*b*	*l*	*c*	*d*	*l*_*g*_	*l*_1_	*w*_1_				
		Best matching	22.31	19.10	7.00	10.36	9.64	6.10	3.34	2.16				
		Size reduction	19.11	14.54	6.61	8.92	8.49	6.02	6.79	2.29				
	Relative		*l*	*c*	*d*	*d*_*l*_	*d*_*w*_	*l*_*gr*_	*l*_1*r*_	*w*_1_				
		Best matching	6.52	10.82	10.41	5.34	4.58	0.28	0.17	2.22				
		Size reduction	7.02	8.61	8.59	4.67	3.10	0.33	0.41	2.29				
II	Absolute		*a*	*b*	*l*	*d*_*l*_	*R*	*R*_1_	*l*_*g*_	*l*_1_	*R*_*h*_	*d*_*r*_	*d*_*c*_	*w*_1_
		Best matching	25.00	19.94	9.58	0.39	5.33	1.50	9.68	4.26	2.54	0.42	1.23	1.11
		Size reduction	21.04	10.73	7.78	0.51	4.30	2.55	8.31	6.00	3.16	0.44	2.35	0.50
	Relative		*l*	*d*_*l*_	*R*	*R*_1_	*d*_*a*_	*d*_*b*_	*l*_*gr*_	*l*_1*r*_	*R*_*hr*_	*d*_*rr*_	*d*_*cr*_	*w*_1_
		Best matching	10.10	0.39	5.40	0.26	2.14	6.15	10.06	0.22	0.21	0.11	0.79	0.63
		Size reduction	9.52	0.26	4.01	0.10	3.41	3.00	9.17	0.47	0.33	0.47	0.54	0.50
III	Absolute		*a*	*b*	*l*	*l*_*p*_	*w*_*p*_	*l*_*s*_	*w*_*s*_	*d*	*l*_*g*_	*r*_*a*_	*r*_*b*_	
		Best matching	30.66	16.27	11.33	13.63	6.56	11.39	0.64	4.05	9.20	1.88	1.57	
		Size reduction	23.07	8.70	13.27	9.64	8.70	9.11	0.76	1.54	9.45	3.06	4.19	
	Relative		*l*_*gr*_	*l*	*l*_*s*_	*w*_*s*_	*d*	*d*_*a*_	*d*_*ls*_	*d*_*ws*_	*d*_*w*_	*r*_*ar*_	*r*_*br*_	
		Best matching	8.78	12.32	10.62	0.20	5.05	5.45	0.69	0.58	3.58	0.24	0.41	
		Size reduction	9.23	11.82	9.87	0.49	5.11	2.49	0.49	0.78	0.73	0.80	0.41

The results allow us to draw several conclusions concerning the performance of the optimization process, depending on the parameterization used. These can be summarized as follows:

- When optimizing for improvement of the in-band matching, relative parameterization is noticeably better (or at least comparable with the absolute one in the case of Antenna I). For Antennas II and III, relative parameterization yields the designs that are better by about 1.5 dB on the average;
- At the same time, repeatability of results is improved for relative parameterization as compared to the absolute one, which is indicated by a generally smaller values of the standard deviation of the objective function;
- When optimizing for minimum size, absolute parameterization is consistently better than the relative one. The improvement is as high as 25 mm^2^ on the average, which is about ten percent in relative terms;
- Repeatability of solutions when optimizing for size reduction is similar for both parameterizations although slightly in favor of the absolute one on the average;
- The average reflection constraint violation (when optimizing for size reduction) is similar for both parameterizations. It should be noted at this point that the level of violation is controlled by the penalty coefficient *β* (cf. (3)), which was set to 10^3^ in all cases. Increasing this value would reduce the average violations while being detrimental to the obtained size reduction^65^.
- The computational cost of the optimization process is consistent for both parameterizations, the differences are statistically insignificant. It can also be observed that the expenses associated with size reduction are higher than those required for matching improvement, which is due to the fact that the former task is a constrained one with nonlinear inequality constraint; thus the problem is numerically more demanding. Also, it can be observed that the computational complexity is more or less proportional to the dimensionality of the parameter space, which is expected for gradient-based trust region procedures with numerical derivatives.

The overall conclusion is that the performance of the optimization process demonstrably depends on the parameterization. Based on the numerical experiments presented in this section, the absolute parameterization is shown to be more suitable for solving size reduction tasks, whereas relative parameterization is more beneficial when optimizing electrical characteristics of the antenna. Here, additional studies will be necessary to investigate the properties of both parameterizations, especially when handling other types of antenna responses (axial ratio, gain, etc.), or carrying out size reduction under multiple constraints. Notwithstanding, the results obtained in this work provide a clear indication of the importance of geometry parameterization in the optimization context. Furthermore, although our studies were conducted for local parameter tuning (here, using a gradient search algorithm), selecting parameterization has further reaching consequences when considering other optimization frameworks. For example, eliminating geometry constraints (both linear and nonlinear) as in relative parameterization facilitates utilization of nature-inspired algorithms for which the ‘natural’ environment is a box-constrained domain.

The reasons for superiority of absolute parameterization over the relative one for matching improvement, and quite the opposite performance for size reduction are not clear at this point. It seems that one of the factors is that the primary objective in size reduction task is a smooth function of geometry parameters, whereas the boundary of the feasible region is determined by the EM-evaluated condition (i.e., inherently noisy). In this setup, the additional constraints pertinent to absolute parameterization do not aggravate the problem because of being smooth as well. On the other hand, absolute parameters give a more direct account for antenna size, which is not the case for relative parameterization. When optimizing for best matching, the latter factor is not as important, whereas the necessity of handling constraints might be detrimental to the quality of the optimization outcome and, therefore, give an edge to the relative parameterization.

Although in this work we focused on two specific optimization tasks: matching improvement and size reduction, a similar analysis can be made for other types of problems. This will be the subject of the future work; however, to emphasize the general importance of parameterization, an additional set of experiments has been performed for Antenna I, which is reduction of in-band realized gain variability. Here, the objective function to be minimized is defined as *U*(***x***) = max{3.1 GHz ≤ *f* ≤ 10.6 GHz : |*G*(***x***,*f*)|} – min{3.1 GHz ≤ *f* ≤ 10.6 GHz : |*G*(***x***,*f*)|}, where *G*(***x***,*f*) is a broadside realized gain at the design ***x*** and frequency *f*. The task is subject to constraint *S*(***x***) ≤ –10 dB, *S*(***x***) = max{3.1 GHz ≤ *f* ≤ 10.6 GHz : |*S*_11_(***x***,*f*)|}. Similarly, as for the size reduction task, it is handled implicitly (cf. (3)) with the penalty function of the form *c*(***x***) = [(*S*(***x***) + 10)/10]^2^; in this case, we use small value of the penalty coefficient to foster the improvement of the primary objective at the expense of tolerating certain violation of the reflection constraint. The reason for considering this particular problem is that stable gain is important for broadband antennas, yet it is difficult to achieve. Numerical optimization turns instrumental here.

**Figure 5:**
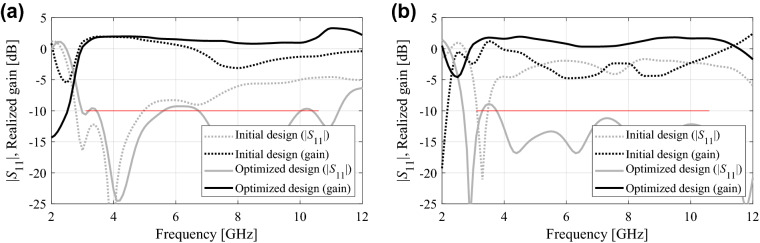
Selected optimization runs for Antenna I oriented towards gain variability reduction (cf. Table 6): initial design (⋅⋅⋅⋅), design optimized for minimum gain variability, (—), **(a)** absolute parameterization (gain variability 1.2 dB), **(b)** relative parameterization (gain variability 1.7 dB). Reflection and realized gain characteristics shown using gray and black lines, respectively. (Matlab R2016a https://www.mathworks.com).

Table 6 provides numerical results in terms of the average in-band gain variability, its standard deviation, as well as the computational cost of the optimization process. Figure 5 shows antenna responses for the selected algorithm runs. The parameter vectors corresponding to the optimized design of Fig. 5 are x^*1^ = [21.21 14.97 6.26 11.04 11.12 4.87 3.73 0.40]^*T*^ (absolute parameterization) and x^*2^ = [7.33 11.06 10.50 1.71 6.70 0.37 0.20 2.40]^*T*^ (relative parameterization). It can be observed that absolute parameterization is more advantageous for handling gain characteristics. The obtained gain variability lower by over 0.7 dB on the average than for relative parameterization; the standard deviation is noticeably smaller as well. At the same time, the computational costs of the optimization process are comparable for both parameterizations, which is consistent with the results contained in Table 4.

**Table 6:** Gain variability minimization results for Antenna I.

Optimization scenario	Performance metric	Parameterization	
		Absolute	Relative
Gain variability minimization	*U*(x*)^1^ [dB]	1.58	2.31
	Std[*U*(x*)] [dB]	0.44	0.69
	Cost^2^	94.0	86.8

^1^Optimized in-band gain variability, averaged over ten algorithm runs.

^2^Number of EM simulations averaged over ten algorithm runs.

## Conclusion

This paper investigated the issue of geometry parameterization in the context of EM-driven parameter tuning of antenna structures. The two types thereof were considered, the absolute (all antenna dimensions expressed in the appropriate length units, e.g., millimetres), and the relative one with the sizes of the structure components expresses in relation the size of encapsulating elements. The qualitative merits of both parameterizations were discussed, followed by comprehensive numerical experiments carried out using three broadband antennas. At the level of the optimization problem formulation, the advantage of absolute parameterization is its simplicity, which comes at the expense of being accompanied by a number of constraints necessary to ensure the geometrical consistency of the structure. On the other hand, relative parameterization eliminates the need for additional constraints making the problem domain as simple as an interval (i.e., parameter space defined using only lower and upper bounds for the parameters).

The numerical results obtained for two design scenarios (optimization for minimum size and best matching) with the optimization executed multiple times from random initial designs, indicate that the performance of the EM-driven design process is affected by the choice of parameterization to a great extent. In particular, absolute parameterization turns out to be more suitable for solving size reduction tasks, whereas relative parameterization is favoured for handling electrical characteristics (here, represented by the in-band matching improvement task). The performance differences are quite significant: the average maximum in-band matching is better by 1.5 dB when using absolute versus relative sizing, whereas relative parameterization leads to size reduction better by 25 mm^2^ (or about ten percent) as compared to the absolute one (again, when averaged over all algorithm runs and all considered antenna structures).

The reasons for these differences seem to be related to whether a particular task is formulated as a constrained or unconstrained one, but also to whether the primary objective is computationally cheap (as in size reduction tasks) or expensive (as when handling antenna electrical characteristics). An additional reason might be related to the overall objective function landscape, which favours absolute parameterization when the goal is related to the physical size of the antenna, yet fosters relative dimension sizing in other cases. More experiments are needed to determine a suitability of particular ways of parameterizing antenna geometry across a broader range of EM-driven design tasks, which will be the subject of the future work.
